# Case report: A case of mast cell leukemia treated with avapritinib: from diagnostic challenge to transplantation

**DOI:** 10.3389/fonc.2026.1746589

**Published:** 2026-01-26

**Authors:** Danilo Giuseppe Faraci, Daniela Caravelli, Fabrizio Carnevale Schianca, Elena Zaniol, Fiorenza Enrico, Carlo Boffano, Carmelo Labate, Greta Sterpi, Alessandro Fornari, Valentina Sangiorgio, Elena Crisà

**Affiliations:** 1 Medical Oncology, Hematopoietic Stem Cells Unit, Turin Metropolitan Transplant Center, Candiolo Cancer Institute, (FPO-IRCCS), Turin, Italy; 2 Department of Medical Oncology, Candiolo Cancer Institute, Fondazione del Piemonte per l'Oncologia - Istituto di Ricovero e Cura a Carattere Scientifico (FPO-IRCCS), Turin, Italy; 3 Collection and Processing Laboratory, Candiolo Cancer Institute, (FPO-IRCCS), Turin, Italy; 4 Hospital Pharmacy, Candiolo Cancer Institute, Fondazione del Piemonte per l'Oncologia - Istituto di Ricovero e Cura a Carattere Scientifico (FPO-IRCCS), Turin, Italy; 5 Radiology Unit, Candiolo Cancer Institute, Fondazione del Piemonte per l'Oncologia - Istituto di Ricovero e Cura a Carattere Scientifico (FPO-IRCCS), Turin, Italy; 6 SC Neurologia, Ospedale Civile Edoardo Agnelli Di Pinerolo, Pinerolo, Italy; 7 Pathology Unit, Candiolo Cancer Institute, Fondazione del Piemonte per l'Oncologia - Istituto di Ricovero e Cura a Carattere Scientifico (FPO-IRCCS), Turin, Italy; 8 Department of Medical Sciences, University of Turin, Turin, Italy; 9 Department of Oncology, Division of Pathology, San Luigi Gonzaga Hospital, Turin, Italy; 10 Division of Hematology U, University Hospital A.O.U. “Città della Salute e della Scienza”, University of Turin, Torino, Italy

**Keywords:** allogeneic stem cell transplantation, avapritinib, diagnostic challenge, mast cell leukaemia, mastocytosis

## Abstract

Systemic mastocytosis (SM) is a rare hematological neoplasm characterized by the proliferation and accumulation of clonal mast cells (MCs) in the skin, bone marrow, and other extracutaneous organs. Mast cell leukemia (MCL) is the rarest and more aggressive subtype of SM, with very poor prognosis and abysmal survival. Treatment strategies are poorly defined and non-standardized, ranging from symptomatic management to KIT-targeted tyrosine kinase inhibitors and, in selected cases, allogeneic stem cell transplantation, currently the only potentially curative chance for MCL patients. Here, we report a case of a 66-year-old woman affected by MCL, highlighting clinical features, diagnostic challenges and complexity, and our treatment choices. Furthermore, we review current literature data and emerging therapeutic approaches, emphasizing the importance of greater clinical awareness and comprehensive multidisciplinary approach to correctly diagnose and treat this condition in order to improve patient outcomes in this challenging disease.

## Introduction

Mast cell leukemia (MCL) is a form of systemic mastocytosis (SM) characterized by leukemic expansion of atypical immature mast cells (MC) accounting for at least 20% of the bone marrow (BM) cell population (1, 2).

MCL is the rarest type of SM: in a series of 2009 Mayo Clinic patients with 342 consecutive SM cases, there were only four patients with MCL, for a prevalence of approximately 1% (3).

The clinical spectrum of SM is highly heterogeneous, and MCL represents the most severe and aggressive form of these diseases, with very poor prognosis (4–6). In the Mayo Clinic cohort, patients with MCL showed survival of only 2 months, compared with 2 years for SM with associated hematologic disorder (SM-AHNMD) and 3.5 years for other aggressive SM (3). More recent data from the European Competence Network on Mastocytosis (ECNM) registry, representing the largest cohort of patients with MCL ever studied (n=92), showed a median overall survival (OS) of 1.6 years (7).

Given the aggressive nature of MCL, it is crucial to maintain a high level of clinical suspicion so that the disease can be diagnosed early and patients can be treated as soon as possible. Although the diagnostic criteria for MCL have been clearly defined by both the World Health Organization (WHO) and the International Consensus Classification (ICC) (2, 8), pathological and molecular features are not yet fully known and the diagnosis can still be challenging, due to the extreme rarity and heterogeneity of the disease (1).

We present here the case of a patient with a complex clinical course that led to a late diagnosis of MCL, failure of first-line therapy, and avapritinib use as bridge to allogeneic hematopoietic stem cell transplantation (aHSCT).

## Case description

### A complex diagnosis

On September 2022, a 66-year-old woman was referred to a medical Center for lymphadenopathies, ascites, and diarrhea. She was Caucasian, lived in a small Piedmontese village, and worked as a farmhand. The patient’s medical history included arterial hypertension, monoclonal gammopathy of unknown significance (MGUS) IgG-kappa, bulbar duodenitis, surgical removal of an intestinal polyp, positivity for tuberculosis (TBC) QuantiFERON test, and monocytosis (>1,500 cells/mL) confirmed by several consecutive blood tests.

She was firstly referred from another Center that started the diagnostic workup of the lymphadenopathy and ascites looking for a lymphoproliferative disease or a possible solid malignancy. Several diagnostic procedures were performed leading to no diagnosis, including 1) a peripheral blood (PB) immunophenotyping showing reduced T- and B-lymphocytes and increased mature-phenotype monocytes; 2) a computerized tomography (CT) scan that revealed left pleural effusion, conspicuous ascites, numerous abdominal lymphadenopathies, and diffuse structural bone remodeling with osteosclerosis; 3) a positron emission tomography (PET) scan showing increased metabolic activity in lymph nodes, bone, gastrointestinal system, and pelvic ascites, with features of dyskaryokinetic disease with high glucose metabolism; 4) a transvaginal ultrasound revealing no pelvic lesions; and 5) an esophago-gastro-duodenoscopy (EGDS) showing gastric micro-polyps and bulbar duodenitis.

Through exploratory laparoscopy, lymphadenopathy biopsies and paracentesis draining 6,600 mL of ascitic fluid were performed. The cytological examination of the ascitic fluid proved negative, and lymph node biopsy was negative for lymphoproliferative disorders.

Thereafter, the patient experienced a worsening of pleural effusion and ascites, and the onset of splenomegaly (longitudinal diameter of 13.5 cm), as confirmed by CT scan. Two further paracenteses (each one draining approximately 7,000 mL of fluid) were performed. Immunophenotypic analysis of the ascitic fluid revealed the presence of lymphocytes (52%) without immunophenotypic alterations, NK cells (25%), and a monocytic-macrophagic population accounting for 37% of the total cells. MC markers were not initially tested as SM was not clinically suspected.

**Figure 1:**
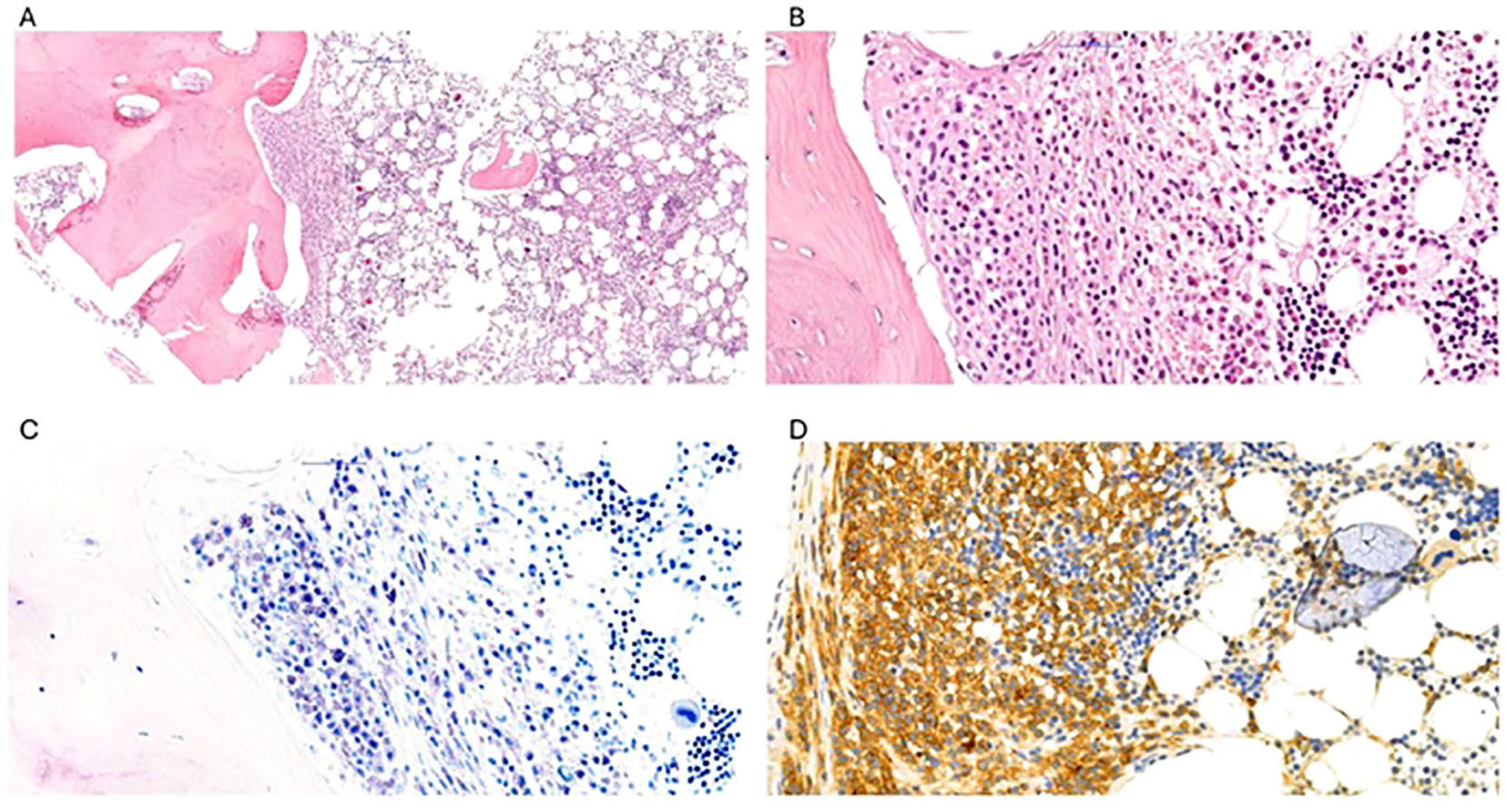
H&E staining of patient’s bone marrow sections (images **(A, B)**, 10× and 40× magnification) showed multifocal dense aggregates of round to spindled atypical cells with an interstitial and paratrabecular distribution. These cells had an abundant eosinophilic cytoplasm with evidence of metachromatic granules on Giemsa staining (image **(C)**, 400× magnification). CD117 immunostaining showed intense and diffuse positivity, confirming the mast cell nature (image **(D)**, 400× magnification). CD30 was also aberrantly expressed on mast cells (not shown).

Given the inconclusive results of all the tests performed, the patient was referred to our Center to be admitted in December 2022; during her hospital stay, a bilateral mammography was performed not revealing any significant abnormalities, and further blood tests evidenced an increase in alkaline phosphatase level [446 U/L]. She also tested negative for Human Herpesvirus 8 (HHV-8). The patient underwent an additional paracentesis (6,200 mL), and a bone marrow (BM) biopsy revealed severe osteosclerosis, fibrosis, and an atypical MC infiltrate (**Figure 1**). At immunohistochemistry, MCs were positive for CD117 and tryptase, with an aberrant expression of CD30. Molecular analysis revealed the presence of the hotspot *KIT* p.D816V mutation, even if we have not available quantitative data, unfortunately.

Following the BM biopsy results, we evaluated the patient’s PB tryptase levels (243 ng/mL). A revision of previously collected abdominal biopsies showed the presence of atypical CD117+, CD25+ MCs. Moreover, the ascitic fluid samples were reevaluated by flow cytometry with the appropriate MC markers and turned out positive for MC infiltration.

To complete the prognostic workup, we performed a PB next-generation sequencing (NGS) analysis, revealing three pathogenetic *TET2* mutations with variant allele fractions (VAFs) of 6.9%, 17.7%, and 38.6%, respectively.

These findings met the WHO and ICC diagnostic criteria for MCL. So in February 2023, the patient was started on midostaurin in addition to the ongoing antihistamines and corticosteroids. Moreover, the patient underwent HLA typing in order to proceed with aHSCT as consolidation therapy, in case of transplantation indication and disease response.

A CT scan performed almost 5 months after the initiation of midostaurin (June 2023) showed a marked reduction in the diffuse ascitic effusion and the disappearance of the left pleural effusion. However, the lymphadenopathies previously detected were unchanged or slightly enlarged; similarly, the diffuse structural bone remodeling showed no improvement compared with the previous examination. A new BM biopsy showed the persistence of a significant MC infiltration (40%), with an improvement of residual physiological hemopoiesis and stromal/bone alterations compared with the previous biopsy. Finally, we observed a progressive increase in tryptase levels (281 ng/mL on August 2023) on midostaurin therapy.

### Avapritinib treatment

Given the unsatisfactory response to midostaurin and the plan to undergo aHSCT, in September 2023 we decided to start a second-line treatment with avapritinib (200 mg/day). The drug was well tolerated by the patient, with no reported side effects.

Treatment with avapritinib resulted in a progressive and significant reduction in tryptase levels from the first month of therapy, which led to normalization of the parameter carried out after 4 months of therapy (35.8 ng/mL after 1 month and 11.8 ng/mL after 4 months).

The CT scan performed 7 months after avapritinib treatment confirmed the ascitic and pleural effusion resolution, along with a reduction in the size of almost all the previously described lymphadenopathies and splenomegaly.

### The allogeneic hematopoietic stem cell transplantation

Since April 2024, the patient began the diagnostic workup required for aHSCT. The results of the BM biopsy performed after 9 months of avapritinib treatment showed a complete response (CR) (hypocellular hematopoietic BM, with mature trilinear hemopoiesis without evidence of MCL). Moreover, at pre-transplant, reevaluation *KIT* mutation was absent. Finally, the pre-transplant NGS reevaluation showed a clearance of one of the three TET2 mutations, but a persistence of the other two with a slightly increased VAF (25.4% vs. 17.7% and 43.1% vs. 38.6%, respectively).

According to latest EBMT Recommendations, patients with AdvSM up to 70 years who have an indication for aHSCT should be considered for transplantation. Our patient failed to achieve an adequate response with first-line *KIT* inhibitor midostaurin, so the TKI switch was our first action. Considering the global restaging of the disease including the CT scan, BM biopsy, and tryptase levels, on second-line therapy, she obtained her best achievable response (partial response) after 9 months of avapritinib treatment. Due to the aggressive initial clinical presentation with “C-findings” and organ damage, we did not consider a bridge-to-transplant polychemotherapy safe and suitable. In addition, no clinical trials were available at that moment. So, given MCL low burden after avapritinib and the presence of a 10/10 HLA-matched unrelated donor, we performed aHSCT without further delay on July 2024. We employed a reduced-intensity conditioning (RIC) with treosulfan 10 g/sqm from day −5 to −3 and fludarabine 50 mg/sqm/day from day −5 to −2 (low TCI score according to EBMT redefined transplant conditioning intensity criteria). Graft-versus-Host disease (GvHD) prophylaxis was performed with post-transplant cyclophosphamide (PTCy) 50 mg/kg/day on days +3/+4, mycophenolate mofetil 45 mg/kg/day, and tacrolimus started on day +5.

The intra-hospital course was characterized by satisfactory engraftment, absence of acute GvHD and significative systemic infections, and the onset of manageable toxicities. The patient was discharged 26 days after aHSCT.

During follow-up, the disease was reevaluated at day +41 with a BM biopsy, tryptase serum levels, and CT scan. BM examination revealed a persistent CR. The serum tryptase level was normal. Lymph node and bone involvement was stable. Moreover, chimerism at days +28, +56, and +85 was full donor.

Unfortunately on day +77, the patient developed acute intestinal GvHD with diarrhea and abdominal pain, poorly responsive to budesonide, rifaxamin, and probiotics administration. So, the initiation of systemic corticosteroids at the dose of 0.5 mg/kg/day was required.

Due to the acute GVHD onset, we decided not to start avapritinib maintenance after aHSCT.

### The neurological complication

The gastrointestinal symptoms were followed by the onset and rapid worsening of psychiatric and neurological symptoms, with an initial depressive syndrome, hypoxia, and weight loss, requiring the start of amisulpride 50 mg/day. Subsequently, a progressive neurological deterioration was observed with the appearance of psychomotor agitation, akathisia, and parkinsonism signs, leading to another admission for a more in-depth diagnostic investigation, in October 2024.

The patient’s neurological status was characterized by several cognitive and motor symptoms: mutism, confusion, delusional ideation, ideomotor slowing, and limb tremors. Neurological investigations included 1) an electroencephalography (EEG) revealing non-specific alterations; 2) a lumbar puncture, negative for detection of neurotropic virus infection, anti-CNS antibodies, or MCs; and 3) a brain MRI revealing a signal alteration in the pontine white matter, with a FLAIR hyperintense signal (faint and with blurred margins), a mildly hypointense baseline T1 signal without significant enhancement, and a mild hyperintensity in the diffusion study (however not presenting florid-phase cytotoxic edema features).

Finally, the definitive neurological assessment led to the diagnosis of central pontine myelinolysis, probably related to an immune-mediated etiology. Once infective causes had been ruled out, treatment with vitamin B1 was started. Due to the possible role of tacrolimus in the pathophysiology of neurological toxicity, it was discontinued, and immunosuppressive (IS) therapy was initiated with methylprednisolone 1 mg/kg/day and ruxolitinib 10 mg twice daily.

No neurological improvements occurred on IS therapy, and both steroids and JAK inhibitor were discontinued after tapering by day +155, in absence of signs and symptoms of GvHD.

Continuous treatment with neurological therapies—including L-dopa and levetiracetam to control motor symptoms—and psychiatric drugs—such as citalopram, delorazepam, and chlorpromazine—were settled. Unfortunately, despite pharmacological therapies, the patient developed an irreversible clinical decline leading to stable subcortical dementia.

Sudden neurological deterioration led to a complete dysphagia, requiring nasogastric tube placement for feeding and drug assumption. Clinical conditions continued to worsen thereafter, and the patient eventually died on April 30th, 2025 (at day +293).

## Discussion

Our case highlights that the MCL diagnostic process is often non-linear, requiring a high level of clinical suspicion and the expertise of a multidisciplinary team including the hematologist, pathologist, allergist, gastroenterology, and radiologist.

**Figure 2:**
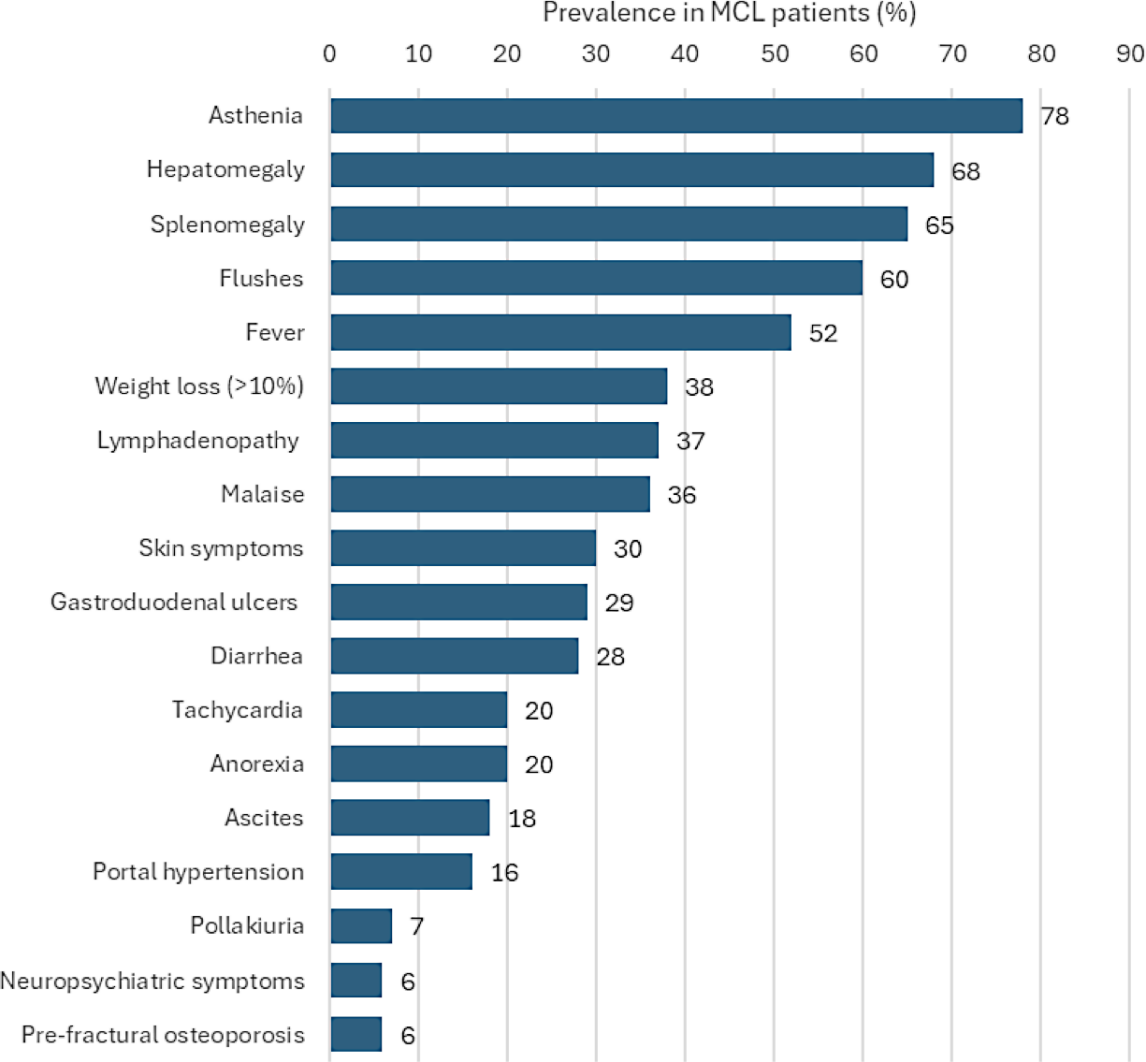
MCL red flags, data from a literature review on 51 MCL patients (9).

Clinical suspicion of MCL must arise in patients with an unexplained disease, in the presence of some “red flags” clinical manifestations (**Figure 2**), such as MC activation symptoms (flushing, fever, malaise, diarrhea, and tachycardia), constitutional symptoms (asthenia, weight loss >10%, and anorexia). Hepatomegaly and splenomegaly can be present in more than 65% of patients, whereas lymph node enlargement (37%) and skin involvement (30%) are two other common symptoms. Gastrointestinal symptoms include gastroduodenal ulcers (29%) often complicated by hemorrhage, ascites (18%, one of our patient’s primary symptoms), and portal hypertension (16%) (5, 9, 10).

It is first necessary to confirm or exclude the diagnosis of SM since MCL is part of the broad spectrum of these diseases, representing the most severe form. For both WHO and ICC 2022, the major diagnostic criterion for SM is the presence of multifocal, dense MC aggregates (each aggregate ≥15 MCs) in BM biopsy and/or other extracutaneous organ (2, 8). Minor diagnostic criteria are as follows:

• >25% spindle-shaped or atypical or immature MCs in BM biopsy/smear or other extracutaneous organs
• Any *KIT* mutation in BM, PB, or other extracutaneous organ
• MCs expressing CD25 ± CD2 (in addition to normal MC markers)
• MCs expressing CD30
• Persistently elevated serum tryptase level (>20 ng/mL) unless there is an associated myeloid neoplasm (in which case this parameter is not valid)

**Figure 3:**
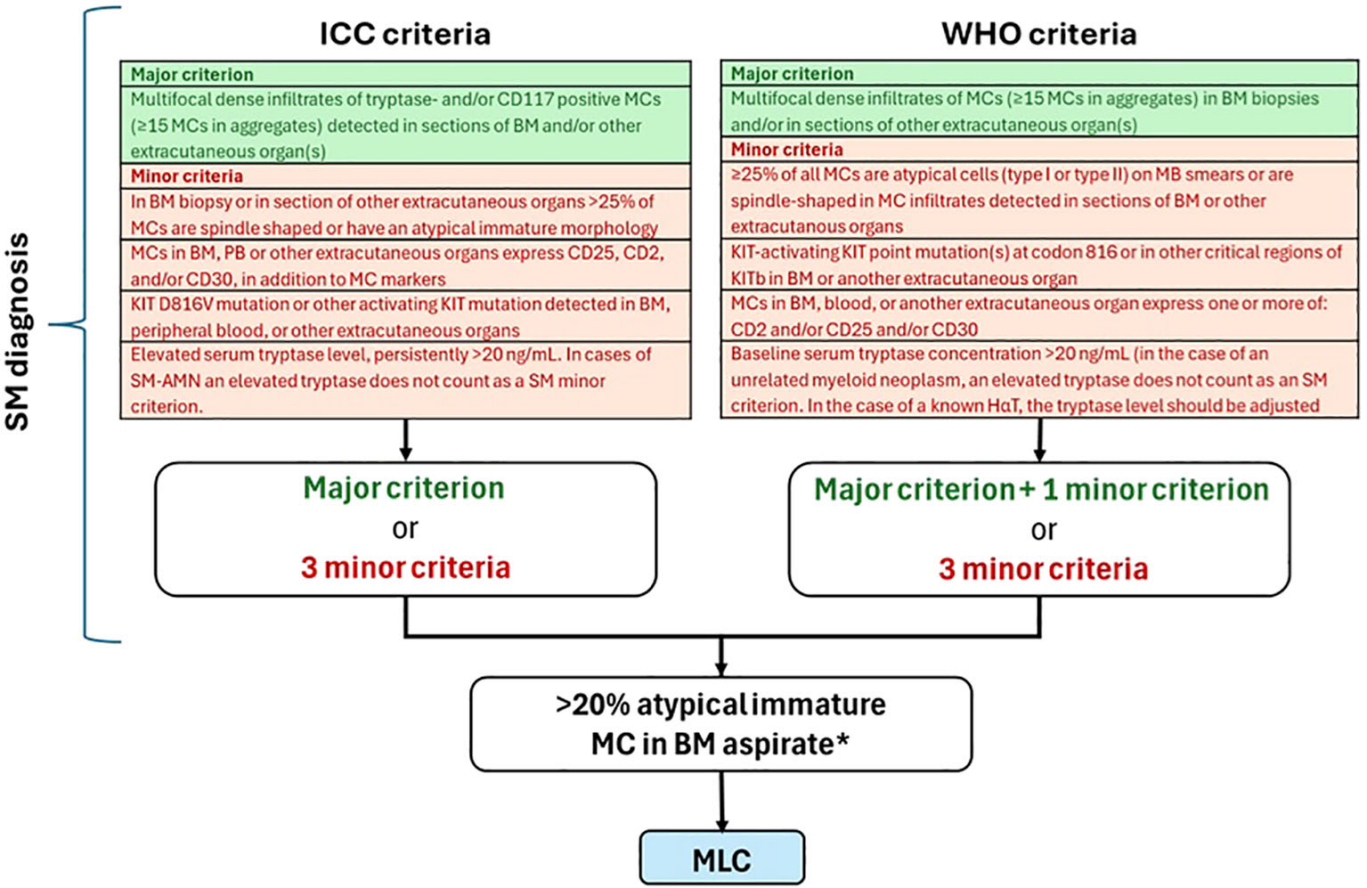
MCL diagnostic flow chart adapted from ICC 2022, WHO 2022, and NCCN 1.2025. * Atypical immature mast cells include promastocytes, metachromatic blast-like forms, and multinucleated or highly pleomorphic mast cells. In the presence of a suboptimal aspirate (dry tap), a BM biopsy showing a dense, diffuse mast cell infiltration of atypical immature mast cells is sufficient to support the diagnosis of mast cell leukemia. BM, bone marrow; MC, mast cell; PB, peripheral blood; SM, systemic mastocytosis.

The WHO diagnostic criteria are slightly more restrictive than ICC ones, as they require the major criterion plus one of the minor criteria or at least three of the minor criteria. According to ICC, the major criterion is sufficient itself for the diagnosis of SM; alternatively, at least three minor criteria must be met (**Figure 3**). MCL is defined by the presence of the diagnostic criteria of SM plus the detection of ≥20% of immature atypical MCs in a BM aspirate (including promastocytes, metachromatic blast-like forms, multinucleated or highly pleomorphic MCs) (2, 11). For ICC 2022, a dense diffuse MC infiltration in BM biopsy is sufficient to support the diagnosis of MCL in cases with inadequate bone marrow aspirates (i.e., “dry tap”) (2, 8).

Moreover, MCL may present as *de novo* disease or as the transformation of advanced systemic mastocytosis (AdvSM), SM-AHNMD, or even indolent systemic mastocytosis (ISM) (10, 12). Furthermore, MCL is very often associated with other hematological neoplasms (up to 71% of cases), e.g., chronic myelomonocytic leukemia (CMML), chronic eosinophilic leukemia (CEL), myelodysplastic syndrome (MDS), and unclassifiable myelodysplastic/myeloproliferative neoplasms (MDS/MPN-U). The presence of a confirmed diagnosis of another hematological neoplasm may delay MCL diagnosis, leading clinical focus in the wrong direction. Therefore, in patients with the previously listed onco-hematological diseases, clinical suspicion for MCL should always be maintained at a high level.

Mutations in the TET2 gene are found in approximately 20%-30% of AdvSM patients, especially loss-of-function ones and frequently alongside the common KIT p.D816V mutation, according to literature data (13–15). This genetic alteration affects epigenetic regulation (DNA methylation), contributing to the aggressive progression of the disease to MCL forms and indicating a poorer prognosis. In our patient, the persistence of TET2 alterations in the presence of KIT mutation clearance throughout treatment may have played the role of driver mutation in the progression of the disease.

**Figure 4:**
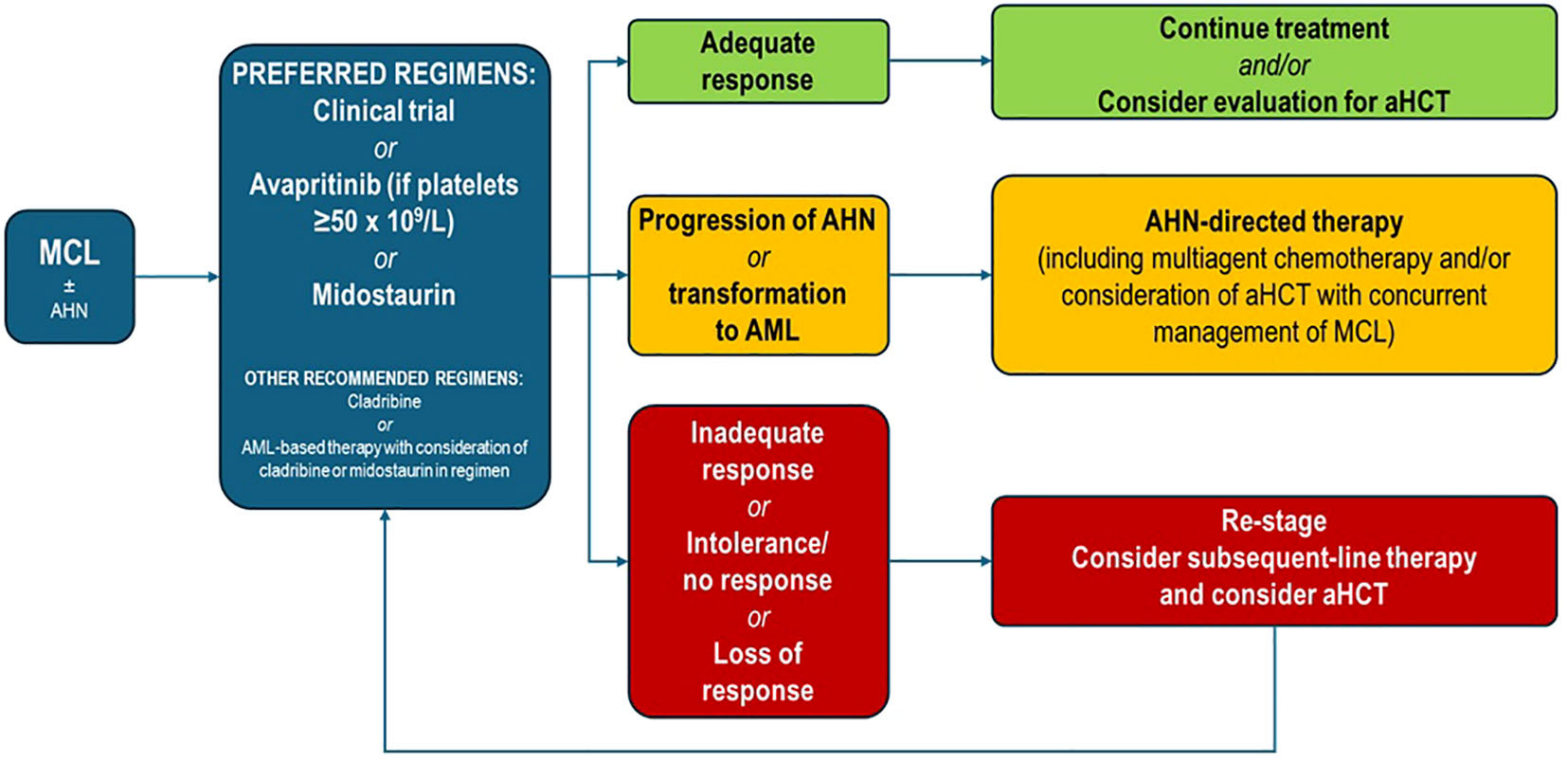
Treatment for MCL ± AHN according to NCCN 1.2025 guidelines. aHCT, allogeneic hematopoietic cell transplant; AHN, associated hematologic neoplasm; AML, acute myeloid leukemia; MCL, mast cell leukemia.

Given the rarity of MCL, treatment is also poorly defined. Several studies and registries attempted to summarize pathological and molecular features as well as survival outcomes in MCL patients (16, 17). There are no evidence-based guidelines for MCL treatment, and information on the efficacy and safety of available drugs is derived from small or retrospective studies and case reports (18). The National Comprehensive Cancer Network (NCCN) SM Diagnosis and Treatment Guidelines represent a summary of current knowledge on MCL management and are summarized in **Figure 4** (12). Referring patients with MCL to specialized centers where they can be treated by a multidisciplinary team is highly recommended. Moreover, the administration of treatments for MC activation symptoms (antihistamines and corticosteroids) is recommended in any patient with SM, since their potential risk of anaphylaxis and training on epinephrine auto-injectors use should be provided (12).

Cytoreductive treatment must be added to this background therapy (12). According to NCCN, avapritinib or midostaurin is the preferred first-line cytoreductive treatment; however, avapritinib is currently approved as a second-line option in Europe since 2022 (19). Midostaurin is an oral multikinase inhibitor with activity against the KIT D816V mutation. In a study of 116 patients with AdvSM, including 16 patients with MCL, treatment with midostaurin 100 mg twice daily resulted in an overall response rate (ORR) of 60%, a median OS of 29 months, and a median progression-free survival (PFS) of 14 months (20). As expected, OS and PFS were shorter in the subgroup of patients with MCL (9 and 11 months, respectively). More recently, data from the ECNM registry in 92 MCL patients showed that midostaurin treatment resulted in a higher median OS than non-treatment (2.3 versus 1.1 years), with the highest benefit when used as first-line treatment (3.2 versus 1.3 years) (7).

Unfortunately, our patient obtained only a partial response to midostaurin. We decided to initiate second-line treatment with avapritinib, a potent selective inhibitor of the KIT D816V mutation (19). In the Phase 1 EXPLORER study, the efficacy and safety of avapritinib were evaluated in 69 patients with AdvSM, 13 of whom had MCL (21). In this study, avapritinib induced profound and durable responses, including molecular remission of KIT D816V, with a favorable tolerability profile. The ORR was 75% in the overall population and 69% in MCL patients. OS at 24 months was 76% in the overall population and 92% in MCL patients, independently of previous midostaurin treatment. In addition, avapritinib resulted in ≥50% reductions in BM MCs and serum tryptase in 92% and 99% of patients, respectively. These results were confirmed by the interim analysis of the Phase 2 PATHFINDER study, conducted in 32 patients with advanced SM (including 4 with MCL) treated with avapritinib 200 mg once daily (22). In this study, avapritinib achieved an ORR of 75% in the overall population. Finally, some emerging real-world data from case series and case reports support the efficacy of avapritinib in the setting of systemic mastocytosis, both in refractory disease and first-line setting (23–26). In our patient, avapritinib enabled to achieve a rapid CR, without evidence of disease in the BM plus normalization of tryptase blood levels and resolution of baseline organomegaly. This allowed the patient to undergo aHSCT, which currently remains the only potentially curative treatment for MCL (27–29). Sriskandarajah et al. analyzed three clinical cases of patients affected by AdvSM-AHN successfully bridged to aHSCT after treatment with avapritinib (30). In addition, retrospective analysis of the German group and new EBMT recommendations for AdvSM highlight how the use of TKI such as avapritinib prior to aHSCT was significantly associated with improved PFS (27, 28). According to data from the DRST/GREM national registries, a response to treatment regimens after aHSCT was achieved in 7/9 of patients (78%) receiving midostaurin and/or avapritinib before aHSCT (27). Furthermore, only four patients out of 30 (13%) were able to start avapritinib as maintenance therapy after aHSCT (obtaining 75% of responses), in line with the challenging post-transplant clinical scenario (27). However, because of advanced age, disease-related organ damage, and inadequate response to chemotherapy, only a small percentage of patients are eligible to transplantation. Moreover, post-transplant outcome is still unsatisfactory. Indeed, in a sample of 57 patients with AdvSM in the pre-TKI era (including 12 with MCL) undergoing aHSCT, a CR was achieved by only 28%, and the 3-year OS was 57% and 17% in the total population and in patients with MCL, respectively (31). This study also highlighted the potential aHSCT-related risks: the 1-year treatment-associated mortality (TRM) was 20% in the total population and 33% in MCL patients. In addition, acute grade 2–4 GvHD events were quite frequent, especially in patients with MCL (42%).

Unfortunately, the case report we have presented exemplifies the possible risk associated with aHSCT, which should be considered before deciding to offer transplantation in the MCL setting. Our patient, in fact, despite obtaining a satisfactory response to aHSCT, experienced a severe neurological event, having a dismal impact on outcome. Neurologic post-transplantation complications can occur frequently, and their etiology is multifactorial (32). Several drugs including cytotoxic agents in the conditioning regimen, TBI, CNIs, and anti-infective drugs may have neurotoxic side effects. In our patient, no TBI or extensive antimicrobial therapy was employed before and during aHSCT, and the onset of neurological symptoms was insidious and subacute. Progressive toxic leukoencephalopathy with central nervous system (CNS) demyelination may represent a rare late fludarabine toxicity. Neurological adverse events related to CNIs typically occur in the early post-transplant period, most commonly presenting as posterior reversible encephalopathy syndrome (PRES). However, the lack of neurological improvement after tacrolimus discontinuation in our patient was not consistent with this hypothesis. In addition, rare persistent neurologic deficits have been reported in literature, such as tacrolimus-related delayed chronic leukoencephalopathy. We ruled out also the rare case of progressive hematological disease with CNS involvement through a lumbar puncture negative for MC detection. Moreover, central and/or peripheral neurological manifestations of GVHD are rare but potentially possible, according to small case report series and reviews. GVHD manifestations are various and represented by immune-mediated encephalitis, cerebrovascular alterations, or demyelinating disease. Diagnosis of neurological manifestations can be highly challenging and remains associated with significant morbidity and dismal prognosis. Obviously, *in vivo* neuropathologic examination is often impossible to perform. We promptly start with the diagnostic neurological workup once the first neurological signs and symptoms were evident in our patient, followed by an accurate neurological specialist evaluation. Finally, prompt therapeutic interventions were implemented, including tacrolimus discontinuation, initiation of immunosuppressive therapy after exclusion of an infectious etiology, and optimization of best supportive care for symptom control. Unfortunately, these measures failed to result in clinical improvement for our patient.

## Conclusions

Diagnosing and managing MCL could be very complex and challenging. Our patient’s clinical course underscores several key lessons. First, early and accurate diagnosis of MCL requires a high index of clinical suspicion, especially in patients with non-specific systemic symptoms and comorbid hematological disorders. The delay in our patient’s diagnosis emphasizes the importance of considering MCL in the differential diagnosis when MC-related symptoms and signs (e.g., ascites and lymphadenopathy) are present. A multidisciplinary approach involving different specialists is essential for navigating the diagnostic landscape of MCL.

Second, treatment decisions for MCL must be individualized and tailored to the clinical response. Our patient’s suboptimal response to midostaurin led to the timely introduction of avapritinib, which produced a biochemical and radiological response, as a bridge to aHSCT—currently the only potentially curative treatment for this condition. The timely access to innovative therapies is of paramount importance, and treatment choices should be aligned with both regional drug approvals and international guidelines. The successful use of avapritinib in our patient before aHSCT underscores its value in managing MCL, especially in the presence of the *KIT* D816V mutation.

Third, although potentially curative, aHSCT has limited success rates in MCL and is associated with a fairly high risk of GvHD and TRM. For these reasons, the benefit/risk balance of aHSCT should be carefully evaluated in patients with MCL.

In conclusion, this case underscores the crucial importance of early recognition, multidisciplinary management, and access to targeted therapies and transplantation to achieve optimal outcomes in MCL. Greater awareness, collaborative care, and further research into MCL pathophysiology and treatment will be crucial for improving the prognosis of this rare yet devastating disease.

## Data Availability

The original contributions presented in the study are included in the article/supplementary material. Further inquiries can be directed to the corresponding authors.
